# Use of Satellite Observations for Long-Term Exposure Assessment of Global Concentrations of Fine Particulate Matter

**DOI:** 10.1289/ehp.1408646

**Published:** 2014-10-24

**Authors:** Aaron van Donkelaar, Randall V. Martin, Michael Brauer, Brian L. Boys

**Affiliations:** 1 Department of Physics and Atmospheric Science, Dalhousie University, Halifax, Nova Scotia, Canada; 2 Harvard–Smithsonian Center for Astrophysics, Cambridge, Massachusetts, USA; 3 School of Environmental Health, University of British Columbia, Vancouver, British Columbia, Canada

## Abstract

Background: More than a decade of satellite observations offers global information about the trend and magnitude of human exposure to fine particulate matter (PM_2.5_).

Objective: In this study, we developed improved global exposure estimates of ambient PM_2.5_ mass and trend using PM_2.5_ concentrations inferred from multiple satellite instruments.

Methods: We combined three satellite-derived PM_2.5_ sources to produce global PM_2.5_ estimates at about 10 km × 10 km from 1998 through 2012. For each source, we related total column retrievals of aerosol optical depth to near-ground PM_2.5_ using the GEOS–Chem chemical transport model to represent local aerosol optical properties and vertical profiles. We collected 210 global ground-based PM_2.5_ observations from the literature to evaluate our satellite-based estimates with values measured in areas other than North America and Europe.

Results: We estimated that global population-weighted ambient PM_2.5_ concentrations increased 0.55 μg/m^3^/year (95% CI: 0.43, 0.67) (2.1%/year; 95% CI: 1.6, 2.6) from 1998 through 2012. Increasing PM_2.5_ in some developing regions drove this global change, despite decreasing PM_2.5_ in some developed regions. The estimated proportion of the population of East Asia living above the World Health Organization (WHO) Interim Target-1 of 35 μg/m^3^ increased from 51% in 1998–2000 to 70% in 2010–2012. In contrast, the North American proportion above the WHO Air Quality Guideline of 10 μg/m^3^ fell from 62% in 1998–2000 to 19% in 2010–2012. We found significant agreement between satellite-derived estimates and ground-based measurements outside North America and Europe (*r* = 0.81; *n* = 210; slope = 0.68). The low bias in satellite-derived estimates suggests that true global concentrations could be even greater.

Conclusions: Satellite observations provide insight into global long-term changes in ambient PM_2.5_ concentrations. Satellite-derived estimates and ground-based PM_2.5_ observations from this study are available for public use.

Citation: van Donkelaar A, Martin RV, Brauer M, Boys BL. 2015. Use of satellite observations for long-term exposure assessment of global concentrations of fine particulate matter. Environ Health Perspect 123:135–143; http://dx.doi.org/10.1289/ehp.1408646

## Introduction

Long-term exposure to fine particulate matter (PM_2.5_) is associated with morbidity and premature mortality (Dockery et al. 1993; Pope et al. 2009). The Global Burden of Disease (GBD) assessment attributed 3.2 million premature deaths per year to ambient PM_2.5_ exposure, such that PM_2.5_ is one of the leading risk factors for premature mortality (Lim et al. 2012). Assessments and indicators of the health effects of long-term exposure to PM_2.5_, such as the GBD assessment, the World Health Organization (WHO) assessment (http://www.who.int/gho/phe/outdoor_air_pollution/burden/en/) and the Environmental Performance Index (http://epi.yale.edu), rely on an accurate representation of both magnitude and spatial distribution of PM_2.5_. Long-term trends in PM_2.5_ concentration can inform whether appropriate steps are being taken to mitigate health and environmental outcomes, and can motivate additional action. Global monitoring can occur from a single satellite as it orbits the earth, minimizing artifacts that may result from regional differences in ground-level network design and operation. Satellites also offer one of the few observationally based sources for long-term PM_2.5_ concentrations that can represent long-term exposure and detect significant changes in many parts the world.

Satellite retrievals of aerosol optical depth (AOD), which provide a measure of the amount of light extinction through the atmospheric column due to the presence of aerosol, have a global data record extending more than a decade. Differing design characteristics between satellite instruments and their retrievals can benefit particular applications. For example, Collection 5 retrievals from the MODIS (Moderate Resolution Imaging Spectroradiometer) instrument (Levy et al. 2007) provide relatively frequent (daily) global observation and accurate AOD over dark surfaces, but are subject to unknown changes in instrument sensitivity with time which could introduce artificial trends. Retrievals from the MISR (Multi-angle Imaging Spectroradiometer) instrument (Diner et al. 2005; Martonchik et al. 2009) require around 6 days for global coverage, but are accurate for both AOD and trend studies based upon comparisons that include AOD measurements from the AERONET (aerosol robotic network) ground-based sun photometer network (Zhang and Reid 2010). SeaWiFS (Sea-viewing Wide Field-of-view Sensor) (Hsu et al. 2013) instrument sensitivity was stable to within 0.13% over its mission, making it applicable for temporal trends (Eplee et al. 2011), but is less accurate over land for absolute AOD compared with MODIS or MISR because of the lack of a mid-infrared channel (Petrenko and Ichoku 2013).

The relationship between AOD and PM_2.5_ depends on aerosol vertical distribution, humidity, and aerosol composition, which are impacted by changes in meteorology and emissions. One technique of relating AOD to near-surface PM_2.5_ uses the ratio of PM_2.5_ to AOD simulated by a chemical transport model. This parameter allows a ground-level PM_2.5_ estimate to be calculated from satellite AOD retrievals. This approach was first demonstrated using the MISR instrument with the GEOS (Goddard Earth Observing System)–Chem chemical transport model (http://www.geos-chem.org) over the United States for 2001 (Liu et al. 2004), and subsequently extended globally for each of the MODIS and MISR instruments for 2001–2002 at a spatial resolution of about 100 km × 100 km (van Donkelaar et al. 2006).

The first long-term mean, global, satellite-derived PM_2.5_ estimates used this technique to combine filtered values from both MODIS and MISR over 2001–2006 at a spatial resolution of about 10 km × 10 km. This data set demonstrated promising agreement with coincident ground-based observations over North America (*r* = 0.77; slope = 1.07) and globally (*r* = 0.83; slope = 0.86) (van Donkelaar et al. 2010). We hereafter refer to this data set as Unconstrained (UC), owing to the unrestricted freedom it gave satellite AOD retrievals to represent the total aerosol column with no influence from the simulated aerosol column.

Improved correlation with ground-based observations for the year 2005 was achieved using Optimal Estimation (OE) (van Donkelaar et al. 2013). OE constrained AOD retrievals from MODIS top-of-atmosphere reflectances based on the relative uncertainties of observational and simulated estimates (van Donkelaar et al. 2013). The PM_2.5_ estimates produced with this data set used vertical profile information from the CALIOP (cloud-aerosol lidar with orthogonal polarization) satellite instrument to inform the relation of column AOD to ground-level concentrations.

Boys et al. (2014) created a time series of PM_2.5_ anomalies by combining AOD from both SeaWiFS and MISR satellite instruments with spatiotemporal information on the PM_2.5_ to AOD relationship from a GEOS–Chem simulation over 1998–2012. In this paper, we extended the OE-based PM_2.5_ estimates to 2004–2010 and combined them with the UC PM_2.5_ values of van Donkelaar et al. (2010) to produce a global, decadal PM_2.5_ data set at approximately 10 km × 10 km, with improved representation of PM_2.5_ over either data set alone. We then applied the temporal variation based upon SeaWiFS and MISR (Boys et al. 2014) to estimate annual global PM_2.5_ estimates and trends over 1998–2012 at 10 km × 10 km resolution.

## Materials and Methods

*Production of satellite-derived estimates*. We first produced a decadal mean PM_2.5_ estimate over 2001–2010. Following Boys et al. (2014), we combined retrievals from SeaWiFS and MISR (see Supplemental Material, “Description of satellite instrumentation”) with time-varying GEOS–Chem (see Supplemental Material, “Description of the GEOS–Chem chemical transport model”) simulated AOD to PM_2.5_ relationships to infer annual variation in PM_2.5_ over 1998–2012 at a spatial resolution of 0.1° × 0.1° (henceforth referred to as SeaWiFS&MISR PM_2.5_). We then extended both OE and UC to cover the temporal range 2001–2010 by applying to each data set the ratio of a coincident SeaWiFS&MISR PM_2.5_ to its decadal mean. We evaluated each extended data set using ground-based PM_2.5_ observations over North America. The global MODIS land-cover type product (MOD12; Freidl et al. 2010) was used to determine the relative weighting of each data set over each land cover type that maximized agreement with ground-level PM_2.5_ observations following van Donkelaar et al. (2013) to produce an initial global combined decadal mean PM_2.5_ estimate.

We subsequently produced a consistent time series of PM_2.5_ over 1998–2012, inclusive. We applied to the initial decadal mean data set the relative temporal variation of SeaWiFS&MISR PM_2.5_ to produce monthly satellite-derived PM_2.5_ estimates over 1998–2012. We calculated absolute annual trends for both data sets using a general least squares regression of 5-month box-car filtered (i.e., median of ± 5 months from the center date), deseasonalized monthly mean values following Zhang and Reid (2010). This approach reduces the impact of any individual season and its relative sampling rate on the overall trend. Confidence intervals (CIs) are based on the integration of Student’s *t*-distribution, and account for autocorrelation. We use an alpha value of 0.05 to define statistical significance. We superimposed these trends to create global annual PM_2.5_ estimates that were consistent in trend with SeaWiFS&MISR and in magnitude with the initial decadal mean. We used a 3-year running median to reduce noise in the annual satellite-derived values. All PM_2.5_ concentrations are given at 35% relative humidity, except for comparisons involving ground-level measurements outside North America, where the 50% standard is adopted for consistency with the ground-level measurements. This difference in standard can increase satellite-derived PM_2.5_ estimates by approximately 10% due to additional water uptake where hydrophilic aerosols, such as sulfate, dominate.

Following Evans et al. (2013), we estimated dust-free and sea salt–free PM_2.5_ concentrations by scaling total satellite-derived PM_2.5_ concentrations by the monthly simulated relative contribution of the remaining species. These scalars were linearly interpolated from the local simulation resolution to 0.1° × 0.1°. We produced satellite-derived PM_2.5_ surface area estimates for interpretation of the dust- and sea salt–free PM_2.5_ estimates following a similar approach as PM_2.5_ mass concentrations, except that the GEOS–Chem model was used to relate AOD to surface area, rather than to mass (see Supplemental Material, “Description of satellite-derived PM_2.5_ surface area”).

*Collection of ground-based observations for evaluation*. We also collected ground-based PM_2.5_ observations over Canada and the United States at locations operational for at least 8 years between 2001 and 2010. We required European sites to be in operation at least 3 years throughout the decade—less time than for North American locations due to the more recent expansion of this regional network. Details of these monitors are given in the Supplemental Material, “Description of ground-level monitor sources from established networks.”

We collected global ground-based PM_2.5_ measurements from published values based on a literature review using the search terms “aerosol” and “PM_2.5_” in the Thomson Reuters Web of Science (http://www.http://thomsonreuters.com/thomson-reuters-web-of-science/), yielding approximately 3,500 results. We selected 541 papers for detailed evaluation from this list and in-publication citations, and found that 342 contained relevant PM_2.5_ observations. We extracted mean PM_2.5_, seasonal variation, city, country, site description, and geocoordinates as available. We approximated geocoordinates using GoogleEarth (https://earth.google.com) and in-reference maps at 70 locations. Geocoordinates were not clear for 110 sites; we assumed measurements occurred within 0.1° of city center. When necessary, we approximated seasonal variation from figures. We considered an observational period every third month as sufficient for annual representation. Where possible, we inferred annual mean concentrations for sites without observations every third month using the relative seasonal variation from nearby published values at distances of up to 1°. We excluded industrial, traffic, and military studies. We combined observational PM_2.5_ values at locations within 0.1°, weighted by their temporal coverage, and used only locations that had at least 3 months of direct observation, for a total of 210 ground-based comparison sites outside of Canada, the United States, and Europe. A complete list of this ground-based database is available online [http://fizz.phys.dal.ca/~atmos/martin/?page_id=140 (“Ground-level PM_2.5_”)] or by contacting the authors.

We evaluated the combined 15-year PM_2.5_ time series from MODIS, MISR, and SeaWiFS (henceforth “combined”) with annual average ground-based PM_2.5_ observations. We conducted the comparison versus PM_2.5_ measurements from ground-based monitors on all days (not only days coincident with satellite observations). We included in the evaluation the 110 global comparison sites from the literature without clearly specified geocoordinates; we conducted evaluations assuming that each ground-based measurement was located at its respective city center and up to 0.1°, or one pixel, away.

Gridded population estimates at 2.5’ resolution from SEDAC (Socioeconomic Data and Applications Center) (2005) at 5-year intervals starting from 1995, are regridded onto 0.1° × 0.1°. Years beyond 2005 are based on projections. We estimated year-specific population densities using linear interpolation.

## Results

**Figure 1:**
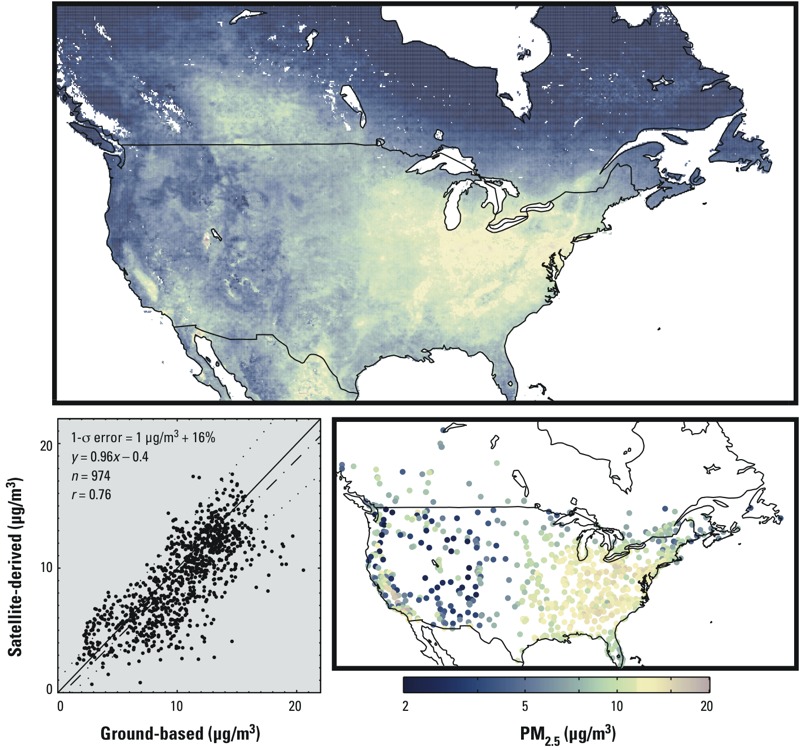
Decadal (2001–2010) mean PM_2.5_ concentrations over North America. White areas denote water or missing values. The top panel displays satellite-derived values. The lower right panel contains averages at ground-based sites in operation at least 8 years during this period. The lower left panel provides a scatterplot and statistics (slope = 0.96; *r* = 0.76; *n* = 974; 1‑σ error = 1 μg/m^3^ + 16%) of the two data sets. The 1:1 line is solid. The line of best fit is dash–dot. The observed 1-σ error is dotted. Ground-based and satellite values are not coincidently sampled to avoid biasing the data toward clear-sky conditions when satellite retrievals occur. Numeric data for GBD regional means are provided in Table 1. A common, logarithmic color scale is used for Figures 1–4.

Figure 1 (top panel) shows decadal mean satellite-derived PM_2.5_ concentrations over North America. Higher concentrations are visible in the eastern United States and in the San Joaquin Valley of California. Figure 1 also shows long-term mean ground-level PM_2.5_ measured during this period over Canada and the United States and comparison with the satellite-derived estimates. Significant overall agreement is found (slope = 0.96, *r* = 0.76; 1-σ error = 1 μg/m^3^ + 16%, where 1-σ error defines the error envelope within which 68% of data points reside). Separate comparisons of OE and UC satellite-derived estimates with the same ground-level monitors gave similar levels of agreement compared with one another (*r* = 0.70–0.71; 1-σ error = 1 μg/m^3^ + 18–20%; not shown). Contributions of OE and UC to the final PM_2.5_ estimates were approximately equal over most land cover types.

**Figure 2:**
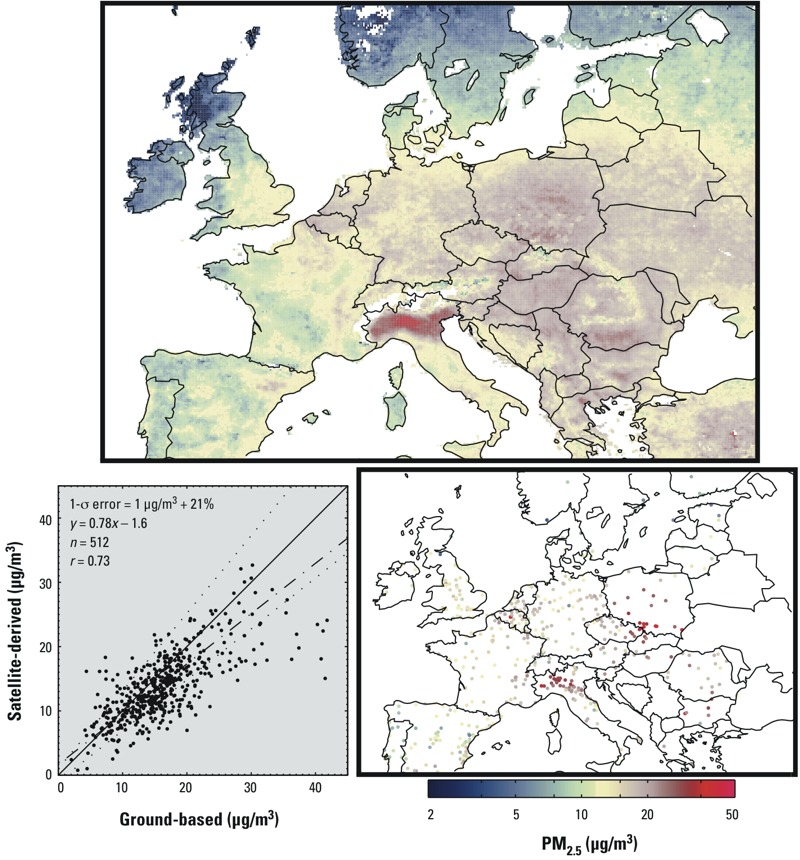
Decadal (2001–2010) mean PM_2.5_ concentrations over Europe. The top panel displays satellite-derived values. The lower right panel contains ground-based values in operation at least 3 years during this period. The lower left panel provides a scatterplot and statistics (slope = 0.78; *r* = 0.73; *n* = 512; 1‑σ error = 1 μg/m^3^ + 21%) of the two data sets, sampled on the same years but noncoincidently on a daily basis. The 1:1 line is solid. The line of best fit is dash–dot. The observed 1-σ error is dotted. Numeric data for GBD regional means are provided in Table 1. A common, logarithmic color scale is used for Figures 1–4.

Figure 2 (top panel) shows decadal mean satellite-derived PM_2.5_ concentrations over Europe. PM_2.5_ is generally higher in Eastern Europe than in Western Europe. The Po Valley in Italy is characterized by the highest regional concentrations, with average PM_2.5_ for some local locations exceeding 35 μg/m^3^ from 2001 through 2010. Figure 2 also shows available long-term mean ground-level observations, which are mostly for the latter part of this period. We find slightly weaker agreement with satellite-derived estimates for Europe than for North America, with slope = 0.78, *r* = 0.73 and 1-σ error = 1 μg/m^3^ + 21%. The weaker agreement likely results from the shorter temporal sampling of 3 years over this region, as illustrated in Supplemental Material, Tables S1 and S2. A cluster of ground-level monitors in southern Poland with annual mean concentrations > 35 μg/m^3^ contributes to the disagreement. PM_2.5_ concentrations in southern Poland near Katowice are higher in wintertime compared with other seasons (Rogula-Kozlowska et al. 2014), when satellite observations are more frequent.

**Figure 3:**
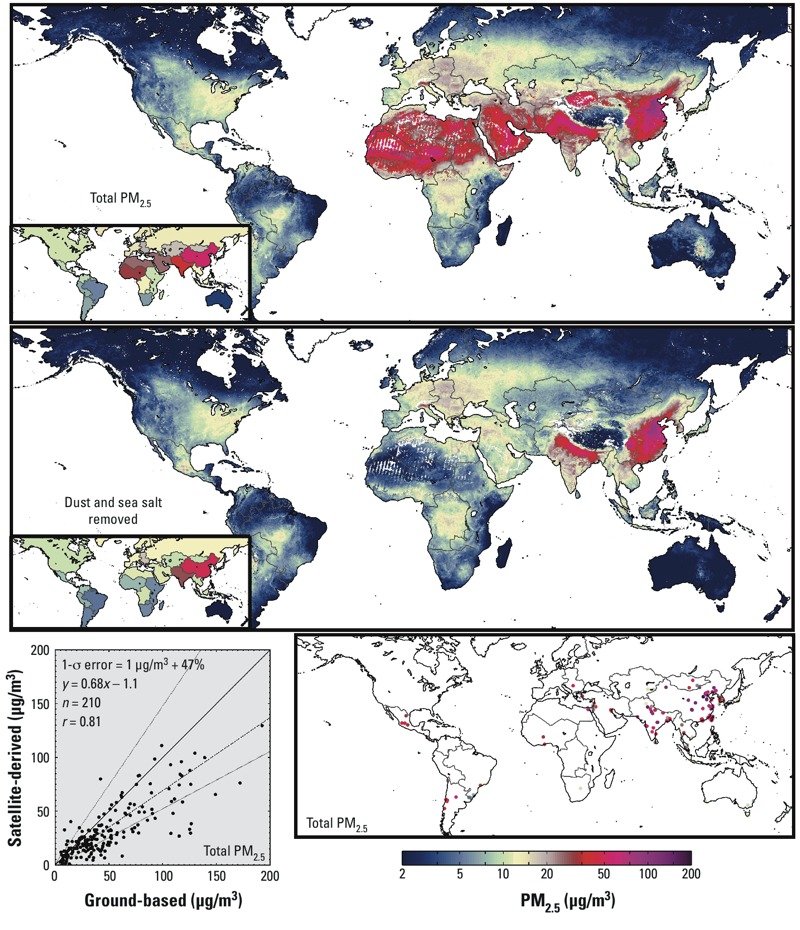
Global decadal (2001–2010) mean PM_2.5_ concentrations. The top panel displays satellite-derived PM_2.5_. The middle panel contains mineral dust– and sea salt–free PM_2.5_. Inset maps display GBD regional population-weighted mean concentrations. Numeric data for GBD regional means are provided in Table 1. The bottom right panel shows the 210 global mean ground-level PM_2.5_ measurements collected from the literature for locations outside Canada, the United States, and Europe. The lower left panel provides a scatterplot and statistics (slope = 0.68; *r* = 0.81; *n* = 210; 1‑σ error = 1 μg/m^3^ + 47%) of the two all-species data sets, sampled on the same years. The 1:1 line is solid. The line of best fit is dash–dot. The observed 1-σ error is dotted. A common, logarithmic color scale is used for Figures 1–4.

Figure 3 (top panel) shows global decadal mean satellite-derived PM_2.5_. PM_2.5_ concentrations in large populated regions of northern India and eastern China, respectively, exceed 60 μg/m^3^ and 80 μg/m^3^. The bottom right panel shows the 210 locations of global mean ground-level PM_2.5_ concentrations outside Canada, the United States, and Europe. Significant agreement (*r* = 0.81) exists, but satellite-derived values tend to be lower than ground-level measurements, with an overall slope of 0.68. Some of this underestimate may arise from locations such as Ulaanbataar, Mongolia, that experience higher concentrations in wintertime and nighttime PM_2.5_ (World Bank 2011) when satellite observations are limited compared with other seasons or daytime. Bias in AOD retrieval may also play a role under the high aerosol loadings found in some regions, such as for MISR AOD over the Indian subcontinent (Dey and Di Girolamo 2010). PM_2.5_ estimates from a sensitivity analysis in which the 110 sites with unspecified geocoordinates were assigned a coordinate at the city center, rather than allowed to shift by up to one pixel from this center, showed similar, but slightly weaker agreement (*r* = 0.78; slope = 0.65).

Table 1 provides a summary of population-weighted satellite-derived exposure according to the regions used by the Global Burden of Disease (Lim et al. 2012). The estimated global population-weighted PM_2.5_ exposure between 2001 and 2010 is 26.4 μg/m^3^ with large spatial variability (SD of 21.4 μg/m^3^). South and East Asia have the highest estimated population-weighted mean exposures, at 34.6 and 50.3 μg/m^3^.

**Table 1:** Population-weighted ambient PM_2.5_ and trend within Global Burden of Disease*^a^* regions.

Region	2001–2010		1998–2012	
	PM_2.5_ (mean μg/m^3^ ± SD)	Dust- and sea salt–free PM_2.5_(mean μg/m^3^ ± SD)	PM_2.5_ trend [μg/m^3^/year (95% CI)]	PM_2.5_ trend [%/year (95% CI)]
Global	26.4 ± 21.4	21.2 ± 19.1	0.55 (0.43, 0.67)	2.1 (1.6, 2.6)
Asia Pacific, high income	16.8 ± 6.4	15.3 ± 6.0	–0.06 (–0.2, 0.08)	–0.4 (–1.2, 0.4)
Asia, Central	17.3 ± 5.7	9.7 ± 3.1	0.29 (0.12, 0.46)	1.7 (0.7, 2.7)
Asia, East	50.3 ± 24.3	45.2 ± 22.5	1.63 (1.09, 2.17)	3.2 (2.1, 4.3)
Asia, South	34.6 ± 15.8	27.8 ± 13.2	1.02 (0.77, 1.27)	2.9 (2.2, 3.6)
Asia, Southeast	11.0 ± 6.4	10.2 ± 6.0	0.30 (0.21, 0.39)	2.7 (1.9, 3.5)
Australasia	3.0 ± 1.0	2.6 ± 0.9	0.01 (–0.02, 0.04)	0.3 (–0.7, 1.3)
Caribbean	7.0 ± 2.5	4.7 ± 1.5	–0.02 (–0.09, 0.05)	–0.3 (–1.3, 0.7)
Europe, Central	17.8 ± 2.6	16.2 ± 2.7	–0.22 (–0.48, 0.04)	–1.2 (–2.7, 0.3)
Europe, Eastern	12.6 ± 3.7	11.2 ± 3.5	–0.04 (–0.25, 0.17)	–0.3 (–2.0, 1.4)
Europe, Western	13.5 ± 4.6	12.1 ± 4.2	–0.25 (–0.37, –0.13)	–1.9 (–2.8, –1.0)
Latin America, Andean	6.6 ± 3.7	6.6 ± 3.7	0.09 (–0.05, 0.23)	1.4 (–0.7, 3.5)
Latin America, Central	8.5 ± 4.3	7.8 ± 4.3	–0.07 (–0.14, 0.00)	–0.8 (–1.6, 0.0)
Latin America, Southern	6.4 ± 2.4	5.4 ± 2.3	0.08 (–0.01, 0.17)	1.3 (–0.1, 2.7)
Latin America, Tropical	5.0 ± 2.6	4.9 ± 2.5	0.01 (–0.03, 0.05)	0.2 (–0.6, 1.0)
North Africa/Middle East	25.5 ± 10.7	11.5 ± 3.6	0.38 (0.17, 0.59)	1.5 (0.7, 2.3)
North America, high income	9.9 ± 3.2	9.6 ± 3.3	–0.33 (–0.41, –0.25)	–3.3 (–4.1, –2.5)
Oceania	2.3 ± 1.1	2.3 ± 1.1	0.09 (0.06, 0.12)	3.9 (2.6, 5.2)
Sub-Saharan Africa, Central	11.4 ± 3.3	9.9 ± 2.7	–0.05 (–0.14, 0.04)	–0.4 (–1.2, 0.4)
Sub-Saharan Africa, East	9.8 ± 8.2	5.5 ± 2.4	0.10 (0.01, 0.19)	1.0 (0.1, 1.9)
Sub-Saharan Africa, Southern	5.9 ± 2.0	5.6 ± 1.9	0.09 (0.01, 0.17)	1.5 (0.1, 2.9)
Sub-Saharan Africa, West	30.8 ± 14.9	7.6 ± 2.9	–0.04 (–0.33, 0.25)	–0.1 (–1.0, 0.8)
^***a***^Lim et al. (2012).				

Figure 3 (middle) presents global estimates of satellite-derived PM_2.5_ with mineral dust and sea salt concentrations removed for 2001–2010. High concentrations remain over southern and eastern China and the Indo-Gangetic Plain. North Africa, the Middle East, and Northwest China have large relative decreases in PM_2.5_, suggesting a large dust component to regional PM_2.5_. North America and Europe show little change in estimated PM_2.5_ resulting from the removal of mineral dust and sea salt. Some studies have suggested that the toxicity of particulate matter is more directly related to particle surface area than to mass (e.g., Maynard and Maynard 2002; Oberdörster et al. 2005). Interestingly, spatial patterns of satellite-derived estimates of PM_2.5_ surface area were similar to spatial patterns of dust-free and sea salt–free PM_2.5_ (see Supplemental Material, Figure S1).

Table 1 summarizes dust- and sea salt–free PM_2.5_ according to GBD region. Dust and sea salt components of PM_2.5_ are responsible for about half the population-weighted decadal mean PM_2.5_ concentrations in Central Asia, North Africa/Middle East, and East sub-Saharan Africa and for three-quarters of the concentration in West sub-Saharan Africa. Dust and sea salt account for 10% of these concentrations in East Asia and 20% in South Asia. Dust and sea salt have little influence over European and North American concentrations.

Table 1 contains population-weighted PM_2.5_ trends over 1998–2012 for each GBD region. A corresponding global trend map following Boys et al. (2014) is in Supplemental Material, Figure S2. Statistically significant increasing population-weighted trends include 1.63 μg/m^3^/year; 95% CI: 1.09, 2.17 (3.2%/year; 95% CI: 2.1, 4.3) over East Asia and 1.02 μg/m^3^/year; 95% CI: 0.77, 1.27 (2.9%/year; 95% CI: 2.2, 3.6) over South Asia. These trends are generally consistent with changes in anthropogenic emissions (Klimont et al. 2013; Kurokawa et al. 2013) and increasing sulfate–nitrate–ammonium concentrations as described in Boys et al. (2014). Trends of 0.38 μg/m^3^/year; 95% CI: 0.17, 0.59 (1.5%/year; 95% CI: 0.7, 2.3) in the Middle East are driven by mineral dust (Chin et al. 2014). Statistically significant downward population-weighted trends include –0.33 μg/m^3^/year; 95% CI: –0.41, –0.25 (–3.3%/year; 95% CI: –4.1, –2.5) over North America and –0.25 μg/m^3^/year; 95% CI: –0.37, –0.13 (–1.9%/year; 95% CI: –2.8, –1.0) over Western Europe. The global population-weighted trend was 0.55 μg/m^3^/year; 95% CI: 0.43, 0.67 (2.1%/year; 95% CI: 1.6, 2.6).

**Figure 4:**
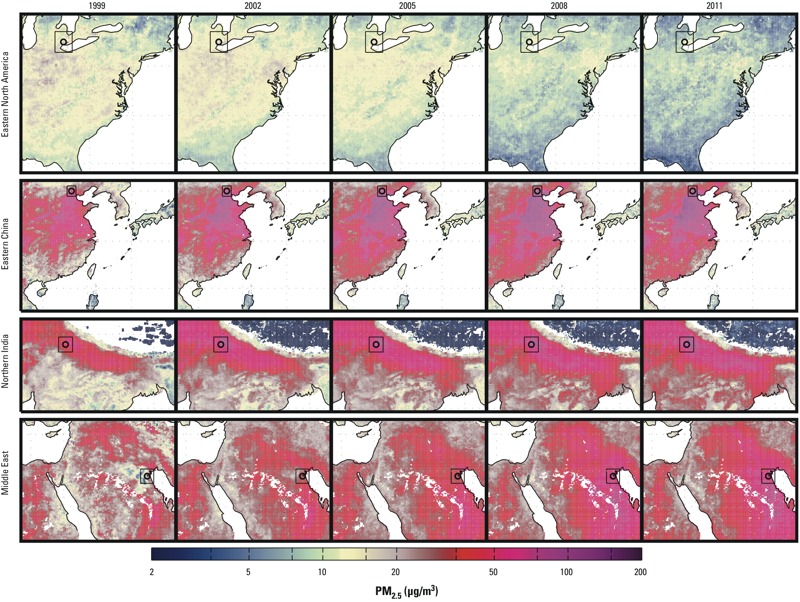
Three-year running mean of satellite-derived PM_2.5_ over sample areas of significant trends. Sub-areas highlighted in Figure 5 are denoted by boxes with black circles around city centers. A common, logarithmic color scale is used for Figures 1–4.

**Figure 5:**
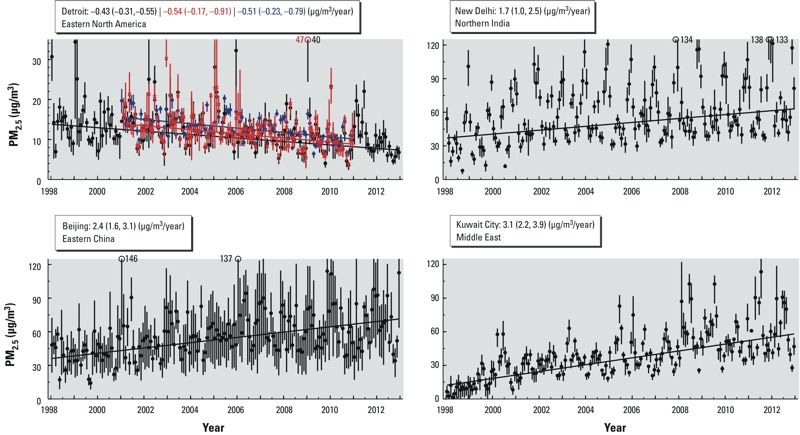
PM_2.5_ time series at the four sub-areas identified in Figure 4. Black dots and vertical lines denote monthly mean and 25th–75th percentile of satellite-derived values. Corresponding ground-level monitor (red x) and satellite-derived coincident with ground-level monitor (blue diamonds) PM_2.5_ are also shown for Detroit in the same notation. Trend and 95% CIs based on these values are provided in the keys. Supplemental Material, Figures S4–S6, overlay satellite-derived PM_2.5_ values with those collected from the literature for Beijing, New Delhi, and Kuwait City.

Figure 4 shows time-series snapshots of PM_2.5_ over the four large-scale areas that demonstrate statistically significant trends. Dust- and sea salt–removed time series over the same regions are shown in Supplemental Material, Figure S3. Changes in PM_2.5_ estimates occur over large spatial domains. Figure 5 shows local trends for a major city within each area. The satellite-derived PM_2.5_ trend estimate for Detroit, Michigan, from 2001 through 2010 (–0.51 μg/m^3^; 95% CI: –0.23, –0.79 was similar to the corresponding trend based on available ground-level observations (–0.54 μg/m^3^/year; 95% CI: –0.17, –0.91). The full 15-year satellite-derived PM_2.5_ time-series changes by –0.43 μg/m^3^/year; 95% CI: –0.31, –0.55, over 1998–2012. Beijing, China, and New Delhi, India, have significant increasing trends over this time period of 2.4 μg/m^3^/year; 95% CI: 1.7, 3.1, and 1.7 μg/m^3^; 95% CI: 1.0, 2.4, respectively, following the regional trends described earlier. Kuwait City has an even larger increasing trend of 3.1 μg/m^3^/year; 95% CI: 2.3, 3.9.

Differences in instrumentation, methodology and site selection inhibit the inference of trends from the PM_2.5_ measurements we collected from published literature and can affect the comparability of these measurements with area-weighted values such as satellite-derived estimates. Comparisons can, however, be informative as shown in the Supplemental Material, Figures S4–S6, which overlay the literature-collected PM_2.5_ for New Delhi, Kuwait City, and Beijing on the satellite-derived estimates from Figure 5. New Delhi measurements such as those by Hyvarinen et al. (2010), taken between 2007 and 2010, suggest a local underestimate in annual mean satellite-derived PM_2.5_ that is driven by wintertime enhancement. Average satellite-derived PM_2.5_ over Kuwait City are within the 31–38 μg/m^3^ range measured by Brown et al. (2008) in 2004–2005. Disparate ground-based measurements in Beijing have a high level of variation with one another, even during similar time periods. For example, Zhang et al. (2007) observed a mean PM_2.5_ concentration of 142 μg/m^3^ at Beijing Normal University from 2001 through 2004, whereas Hopke et al. (2008) observed annual means of 28–42 μg/m^3^ during a similar period of 2002–2004 at urban and suburban locations. Satellite-derived PM_2.5_ are more consistent with the lower range of available measurements in Beijing.

**Figure 6:**
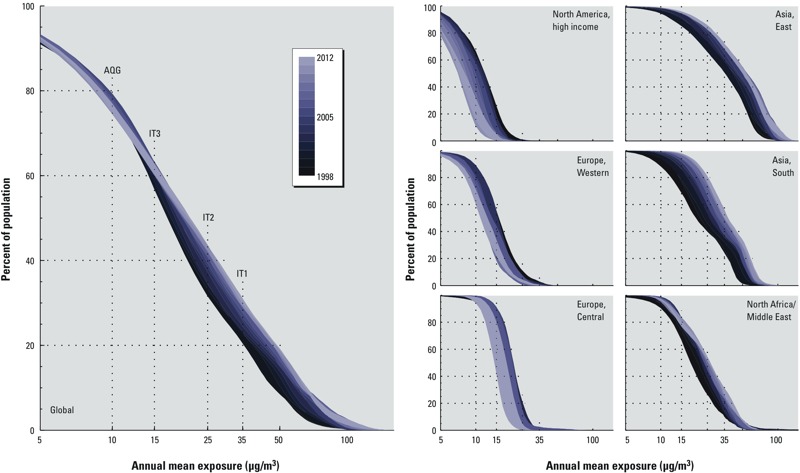
Cumulative distribution of regional annual mean PM_2.5_ for 1998–2012. AQG, IT3, IT2, and IT1 refer to the WHO air quality guidelines of 10, 15, 25, and 35 μg/m^3^.

Figure 6 gives the cumulative distribution of estimated global annual mean PM_2.5_ as a function of time, and for the three GBD regions with the greatest positive and negative trend magnitudes, respectively. Table 2 provides the percent of population living in areas where concentrations are above the WHO interim targets (IT3, IT2, and IT1) and air quality guideline (AQG) for 1998–2000 and 2010–2012 for all regions. A small population-weighted global improvement (1%) of those living within the AQG was estimated for 1998–2012, driven predominantly by improvements to air quality in North America that reduced the population exposed to PM_2.5_ > 10 μg/m^3^ from 62% to 19%. Globally, we estimated that exceedance of IT1 (35 μg/m^3^) rose by 8% over the same time period, reaching 30% by 2010–2012 as driven by increasing PM_2.5_ concentrations in the heavily populated regions of South and East Asia. Because satellite-based values appear to underestimate concentrations measured by ground-based monitors, it is possible that the proportion of populations living above WHO targets could be higher.

**Table 2:** Percent of population (%) in excess of WHO PM_2.5_ target within Global Burden of Disease^*a*^ regions.

Region	AQG (10 μg/m^3^)			IT3 (15 μg/m^3^)			IT2 (25 μg/m^3^)			IT1 (35 μg/m^3^)		
	1998–2000	2010–2012	2010–2012*	1998–2000	2010–2012	2010–2012*	1998–2000	2010–2012	2010–2012*	1998–2000	2010–2012	2010–2012*
Global	76	75	75	57	61	60	32	43	42	22	30	30
Asia Pacific, High Income	77	80	80	50	50	49	9	11	10	1	0	0
Asia, Central	78	84	82	56	69	68	14	18	17	2	2	2
Asia, East	95	99	99	86	95	95	67	84	84	51	70	70
Asia, South	92	100	100	75	98	97	43	78	77	27	52	51
Asia, Southeast	42	55	56	23	27	28	6	7	7	3	2	2
Australasia	0	0	0	0	0	0	0	0	0	0	0	0
Caribbean	15	27	24	2	2	2	1	0	0	0	0	0
Europe, Central	96	96	97	80	63	63	10	3	3	1	0	0
Europe, Eastern	66	68	67	28	22	21	2	0	0	0	0	0
Europe, Western	84	66	66	45	27	26	7	3	3	1	0	0
Latin America, Andean	23	26	26	10	4	4	1	0	0	0	0	0
Latin America, Central	43	34	34	24	9	9	11	1	0	6	0	0
Latin America, Southern	8	8	8	2	1	1	0	0	0	0	0	0
Latin America, Tropical	15	6	6	2	0	0	0	0	0	0	0	0
North Africa/Middle East	93	97	97	72	80	79	35	53	51	15	28	27
North America, High Income	62	19	20	17	2	2	1	0	0	0	0	0
Oceania	0	1	0	0	0	0	0	0	0	0	0	0
Sub-Saharan Africa, Central	65	60	59	34	27	26	5	2	2	1	0	0
Sub-Saharan Africa, East	32	38	38	19	19	20	8	9	9	3	3	3
Sub-Saharan Africa, Southern	3	8	7	0	0	0	0	0	0	0	0	0
Sub-Saharan Africa, West	97	96	95	91	84	84	74	56	55	51	32	32
^***a***^Lim et al. (2012). *Percent of population in excess of target based on 2010–2012 PM_2.5_ concentrations, but using 1998–2000 population distribution. Other columns use a population distribution according to their respective years.												

Table 2 also shows the effect of population change on WHO target achievement as represented by applying a 1998–2012 population distribution on 2010–2012 PM_2.5_ concentrations. This effect, taken as the percent difference between 1998–2000 and 2010–2012 achievement that occurs from population changes, is < 25% across all targets for all regions, and < 10% in most cases. The number of people living above the AQG in some regions has increased due to population changes, accounting for about a quarter of the change seen in Central Asia and South sub-Saharan Africa from 1998 to 2012. About half the change in Eastern Europe is attributable to population, although the overall change is small (2%). Population changes contributed to small reductions in population-weighted mean PM_2.5_ concentrations for regions such as Southeast Asia and North America.

## Discussion

A broad community requires globally consistent estimates of long-term PM_2.5_ exposure and changes over time. For example, this information is used for Global Burden of Disease assessments (Brauer et al. 2012; Lim et al. 2012; WHO 2014), for environmental performance indicators (Environmental Performance Index 2014), and for epidemiologic studies of air pollution health effects at global (Anderson et al. 2012; Fleischer et al. 2014) and regional (Chudnovsky et al. 2013; Crouse et al. 2012; Vinneau et al. 2013) scales. Satellite retrievals offer the most globally complete observationally based data source of this information, but improvements to these estimates are needed to reduce uncertainties.

In this work, we combined the attributes of several recent satellite-derived PM_2.5_ data sets to improve the accuracy in estimates of long-term exposure and changes in annual concentrations from 1998 through 2012. We inferred decadal mean PM_2.5_ from Unconstrained (van Donkelaar et al. 2010) and Optimal Estimation (van Donkelaar et al. 2013) based approaches using the MODIS and MISR instruments. We then applied the relative temporal variation from SeaWiFS and MISR observations (Boys et al. 2014) to represent the annual variation over 15 years. The resultant combined data set had significant agreement with ≥ 8-year means of ground-based observations over North America (slope = 0.96; *r* = 0.76; 1-σ error = 1 μg/m^3^ + 16%) and ≥ 3-year means over Europe (slope = 0.78; *r* = 0.73; 1-σ error = 1 μg/m^3^ + 21%) in noncoincident comparisons that represent both retrieval- and sampling-induced uncertainties. This performance was better than for any of the individual data sets. The agreement between satellite-derived and ground-based PM_2.5_ was higher when limited to coincident samples (i.e., when monitor and satellite data were restricted to only those days when the other was available, the approach used by many previous studies) compared with data not restricted in this manner (as in the present analysis). For example, the correlation of *r* = 0.77 over North America for 2001–2006 previously given by van Donkelaar et al. (2010) drops to *r* = 0.70 when unrestricted by instrumental co-sampling. The unrestricted comparisons used in this present work include any residual effect of satellite sampling on its long-term mean PM_2.5_ estimates and therefore offer a better representation of uncertainty.

A major challenge in evaluating global satellite-derived PM_2.5_ is the paucity of ground-based measurements. We collected a global data set of 210 ground-based observations from the literature and used them to evaluate global satellite-derived PM_2.5_ estimates, including many locations in India and China. Significant agreement was found (*r* = 0.81), although these monitors revealed that satellite-derived PM_2.5_ is typically lower than ground-based observations (slope = 0.68). This underestimate may result from factors such as AOD bias in the MISR retrieval over South and East Asia (Kahn et al. 2009), missing satellite observations during wintertime and/or nighttime if PM_2.5_ concentrations are relatively high at these times (e.g., Katowice, Poland, and Ulaanbaatar, Mongolia), or coarse resolution of either the satellite-derived product or the simulation used to relate AOD to PM_2.5_, which may obscure localized features. The potential underestimate in satellite-derived PM_2.5_ outside North America and Europe furthermore suggests that true PM_2.5_ concentrations may be even greater than we estimated.

Uncertainty in satellite-derived PM_2.5_ decreases with increased sampling and can vary by season. As a result, the satellite-derived PM_2.5_ estimates presented here are best used on large regional scales over multiple years. Studies interested in seasonal variation and/or smaller spatial scales would benefit from some degree of local validation, as available.

We found that decade-long population-weighted ambient PM_2.5_ concentrations estimated for East Asia were nearly double the estimated global mean of 26.4 μg/m^3^, and increased at an annual population-weighted rate of 1.63 μg/m^3^/year; 95% CI: 1.09, 2.17 (3.2%/year; 95% CI: 2.1, 4.3) between 1998 and 2012. Population-weighted concentrations estimated for western Europe and North America over the same period changed by –0.25 μg/m^3^/year; 95% CI: –0.37, –0.13 (–1.9%/year; 95% CI: –2.8, –1.0) and –0.33 μg/m^3^/year; 95% CI: –0.41, –0.25 (–3.3%/year; 95% CI: –4.1, –2.5), respectively, in contrast with increases over South Asia (1.02 μg/m^3^/year; 95% CI: 0.77, 1.27; 2.9%/year; 95% CI: 2.2, 3.6) and the Middle East (0.38 μg/m^3^/year; 95% CI: 0.17, 0.59; 1.5%/year; 95% CI: 0.7, 2.3). Satellite-derived estimates suggest that 30% of the global population lived in regions above the WHO IT1 standard (35 μg/m^3^) for PM_2.5_ in 2010–2012, up from 22% in 1998–2000. We found that most of the changes in exposure were driven by changes in PM_2.5_ rather than changes in population itself.

Both the satellite-derived PM_2.5_ estimates created in and ground-level observations collected for this study are freely available as a public good on our website (http://fizz.phys.dal.ca/~atmos/martin/?page_id=140), the SEDAC website (http://sedac.ciesin.columbia.edu/), or by contacting the authors.

Further developments to satellite retrievals and simulated aerosol profiles will continue to allow improved representation of global exposures to PM_2.5_. In particular, higher resolution satellite retrievals may better capture intraurban variation (Chudnovsky et al. 2013). Recent improvements to MODIS instrument calibration (Levy et al. 2013) may provide an additional data source for trends. Additionally, assessment of trends would benefit from better availability of longer time series of ground-level monitoring data.

## Supplemental Material

(2.5 MB) PDF
