# Supplementary material for: Use of Satellite Observations for Long-Term Exposure Assessment of Global Concentrations of Fine Particulate Matter
Source: Environ Health Perspect. 2014 Oct 24;123(2):135–43. doi: 10.1289/ehp.1408646 (PMC4314252; doi:10.1289/ehp.1408646)
Supplement: (2.5 MB) PDF [file ehp.1408646.s001.508.pdf]

Environmental Health Perspectives doi:10.1289/ehp.1408646

Supplemental Material

Use of Satellite Observations for Long-Term Exposure Assessment of Global Concentrations of Fine Particulate Matter

Aaron van Donkelaar, Randall V. Martin, Michael Brauer, and Brian L. Boys

Description of satellite instrumentation

As described in van Donkelaar et al. (2010; 2013), the Unconstrained (UC) and Optimal Estimation (OE) $PM_{2.5}$ datasets use data from the MODIS (MODerate resolution Imaging Spectroradiometer) instruments. UC used MODIS onboard the Terra satellite, while OE used MODIS onboard both Terra and Aqua. Both MODIS instruments provide near-daily global AOD coverage in the absence of clouds from a polar orbiting, sun-synchronous orbit. Quality assured collection (version) 5 MODIS AOD at 10 km × 10 km over land (Levy et al. 2007) has been validated such that at least two-thirds of its retrievals are within ±(0.05 + 15%) using Aerosol Robotic Network (Holben et al. 2001) measurements of AOD (Remer et al. 2008). Concerns have been raised about drift in MODIS collection 5 over land (Zhang and Reid 2010). We used this dataset only for long-term averages (not trends).

The MISR (Multi-angle Imaging SpectroRadiometer) instrument onboard the Terra satellite is used for the UC dataset (van Donkelaar et al. 2010) and trends (Boys et al. 2014). MISR observes radiation leaving the top of the atmosphere in four spectral bands (0.446, 0.558, 0.672 and 0.866 μm), each at nine viewing angles (±70.5º, ±60.0º, ±45.6º, ±25.1º and nadir). MISR typically takes 6 to 9 days for complete global in the absence of clouds. The MISR AOD retrieval algorithm at 17.6 km × 17.6 km (Diner et al. 2005; Martonchik et al. 2002; Martonchik et al. 2009) has been validated such that two-thirds of retrievals fall within the maximum of ±(0.05 or 20%) of ground truth observations (Kahn et al. 2005), and has reliable trend information over land (Zhang and Reid 2010).

The SeaWiFS (Sea-viewing Wide Field-of-view Sensor) instrument provides near-daily global coverage at 8 wavelengths from a sun-synchronous orbit. The Deep Blue algorithm has recently

been applied to SeaWiFS AOD retrieval at a resolution of 13.5 km (Hsu et al. 2013), providing a well-calibrated retrieval of global AOD from 1998-2010 suitable for trend studies (Hsu et al. 2012). High quality SeaWiFS AOD has been validated such that at least two-thirds of retrievals are within ±(0.05 + 20%) (Sayer et al. 2012).

Daily AOD retrievals from each instrument are regridded onto a regular global 0.1° × 0.1° grid using an area-weighted average prior to relating to $PM_{2.5}$.

Description of the GEOS-Chem chemical transport model

The GEOS-Chem chemical transport model (http://geos-chem.org) solves for the spatial and temporal evolution of atmospheric aerosol and gaseous compounds using meteorological data sets, emission inventories, and equations that represent the physics and chemistry of the atmosphere. We used GEOS-Chem to relate AOD to $PM_{2.5}$ mass and surface area, and to provide prior estimates with which to constrain the OE satellite retrievals of AOD.

Detailed simulation descriptions are contained within the corresponding publications for UC (van Donkelaar et al. 2010), OE (van Donkelaar et al. 2013) and SeaWiFS&MISR (Boys et al. 2014). A major distinction between these simulations are the assimilated meteorological fields used for UC (GEOS-4), OE (GEOS-5) and SeaWiFS&MISR (MERRA). All fields were provided by the Goddard Earth Observing System and represented current versions of available meteorology at the original time of each publication. All simulations were performed globally at 2° × 2.5°. OE additionally used three nested 1/2° × 2/3° regions overs North America, Europe and eastern Asia.

All simulations share a similar treatment of aerosol that include the sulphate-ammonium-nitrate-water system (Park et al. 2004), primary carbonaceous aerosols (Park et al. 2003), secondary

organic aerosols (Henze et al. 2008), sea salt (Alexander et al. 2005), and mineral dust (Fairlie et al. 2007).

Description of satellite-derived $PM_{2.5}$ surface area

AOD is more directly related to $PM_{2.5}$ surface area than $PM_{2.5}$ mass since light extinction is proportional to particle surface area (not volume) and surface area does not require assumptions about particle densities. Satellite-derived estimates of surface area can, therefore, be readily created following the approaches established for $PM_{2.5}$ mass. We produced such estimates of surface area by applying to satellite (MODIS, MISR and SeaWiFS) GEOS-Chem simulations of coincident AOD to ground-level surface area of particles with aerodynamic diameter smaller than 2.5 μm which we refer to as $PM_{2.5}$ surface area. OE, UC, and SeaWiFS&MISR-based surface area was produced using the simulations and methods described for $PM_{2.5}$ in van Donkelaar et al. (2013) and van Donkelaar et al. (2010), respectively, and combined following the approach outlined in the main manuscript. Figure S1 shows the resultant decadal mean $PM_{2.5}$ surface area for comparison with Figure 4.

Description of ground-level monitor sources from established networks

Established $PM_{2.5}$ networks provide a robust source of evaluation for satellite-derived $PM_{2.5}$ concentrations due to their long-term observation period and consistent measurement practices. Ground-level Canadian $PM_{2.5}$ observations were obtained from the National Air Pollution Surveillance network (NAPS; http://www.etc.cte.ec.gc.ca/NAPS/index_e.html), excluding industrial sites. American observations were taken from sites of the Interagency Monitoring of Protected Visual Environments network (IMPROVE;

http://vista.cira.colostate.edu/improve/Data/data.htm) and from the Environmental Protection Agency Air Quality System that employ the Federal Reference Method (FRM; http://www.epa.gov/air/data/index.html). $PM_{2.5}$ measurements at background sites from the European air quality database (Airbase; http://acm.eionet.europa.eu/databases/airbase/) and European Monitoring and Evaluation Programme (EMEP; Torseth et al. 2012) were used over Europe.

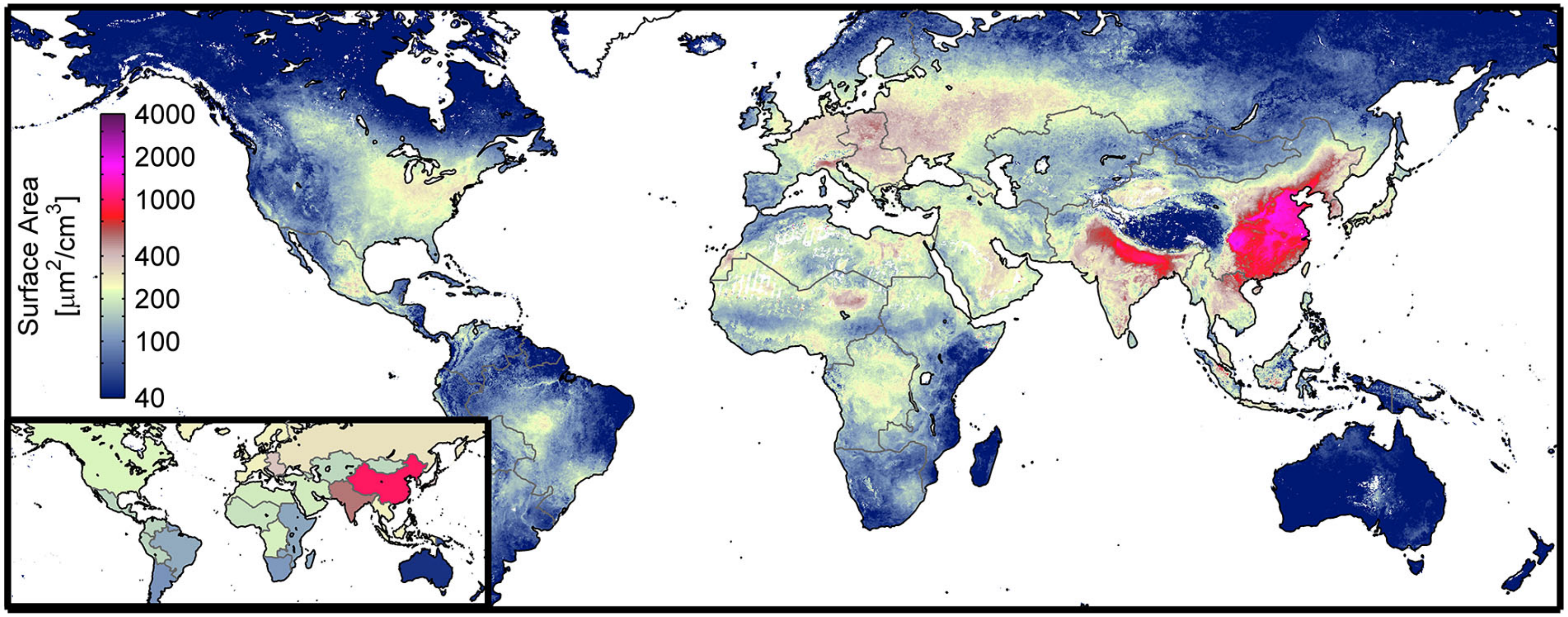

Figure S1. Global decadal (2001-2010) mean $PM_{2.5}$ surface areas. The inset map displays GBD regional population-weighted mean surface area. The logarithmic color scale follows that used for Figures 1-4.

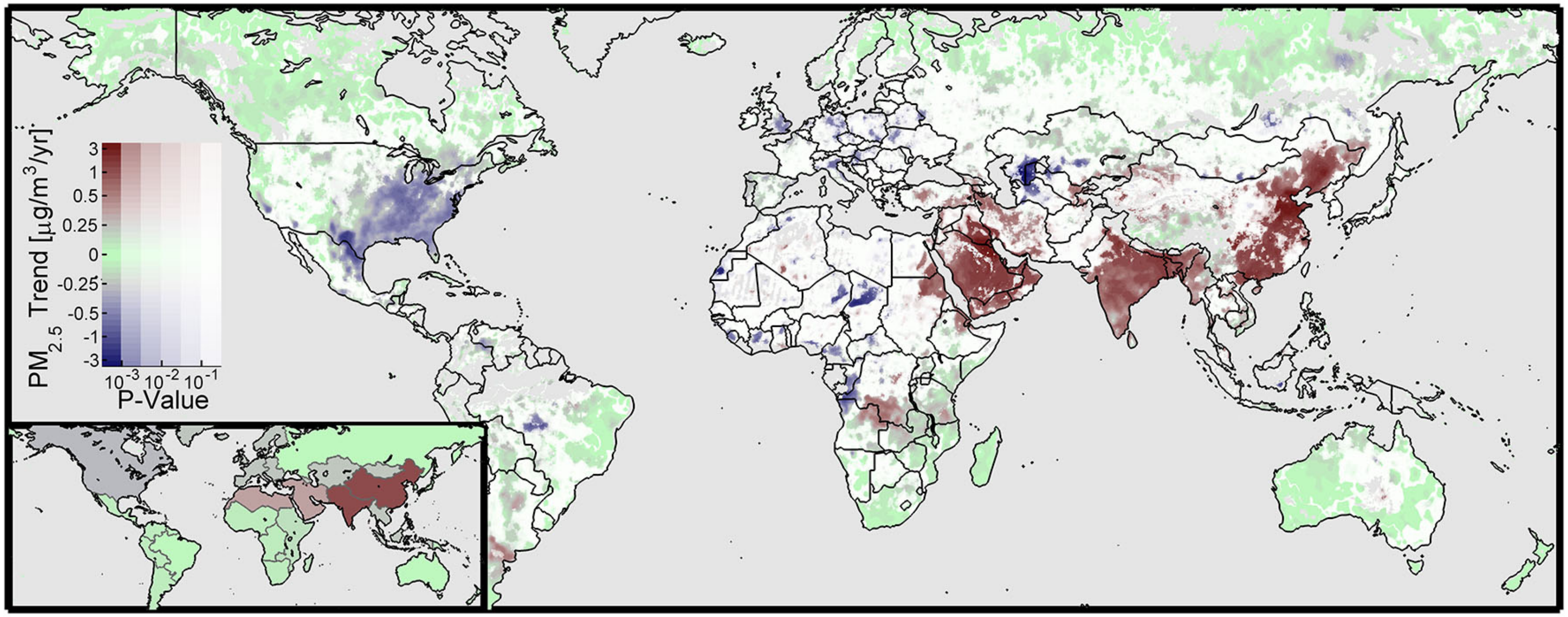

Figure S2. $PM_{2.5}$ annual trend over 1998-2012. The intensity of the colorscale provides a measure of statistical significance. Inset gives population-weighted mean values within GBD-defined regions. Grey areas denote water or missing data.

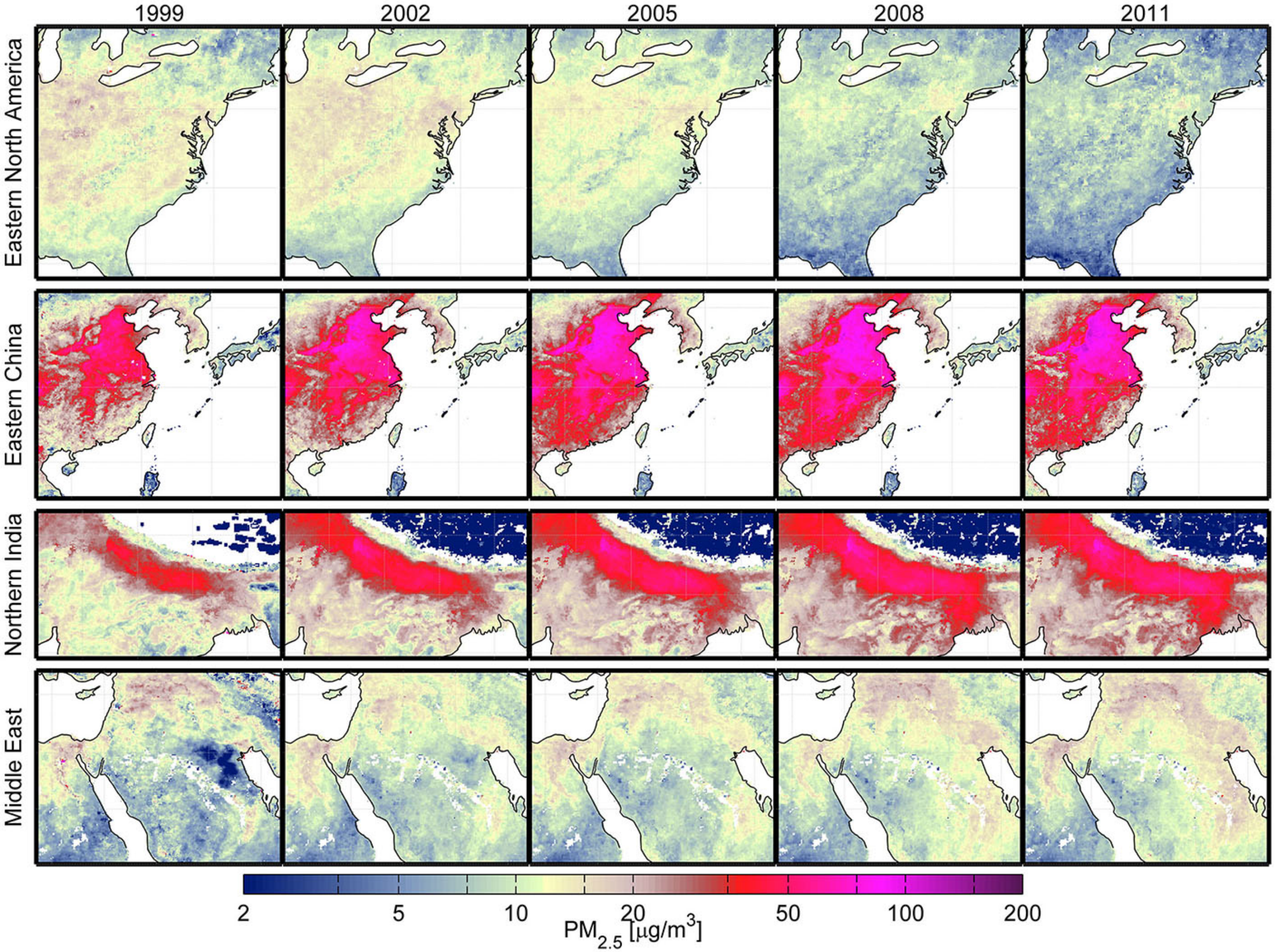

Figure S3. Three-year running mean of satellite-derived dust-and-seasalt-removed $PM_{2.5}$ over sample regions of significant trends. A common, logarithmic color scale is used for Figures 1-4.

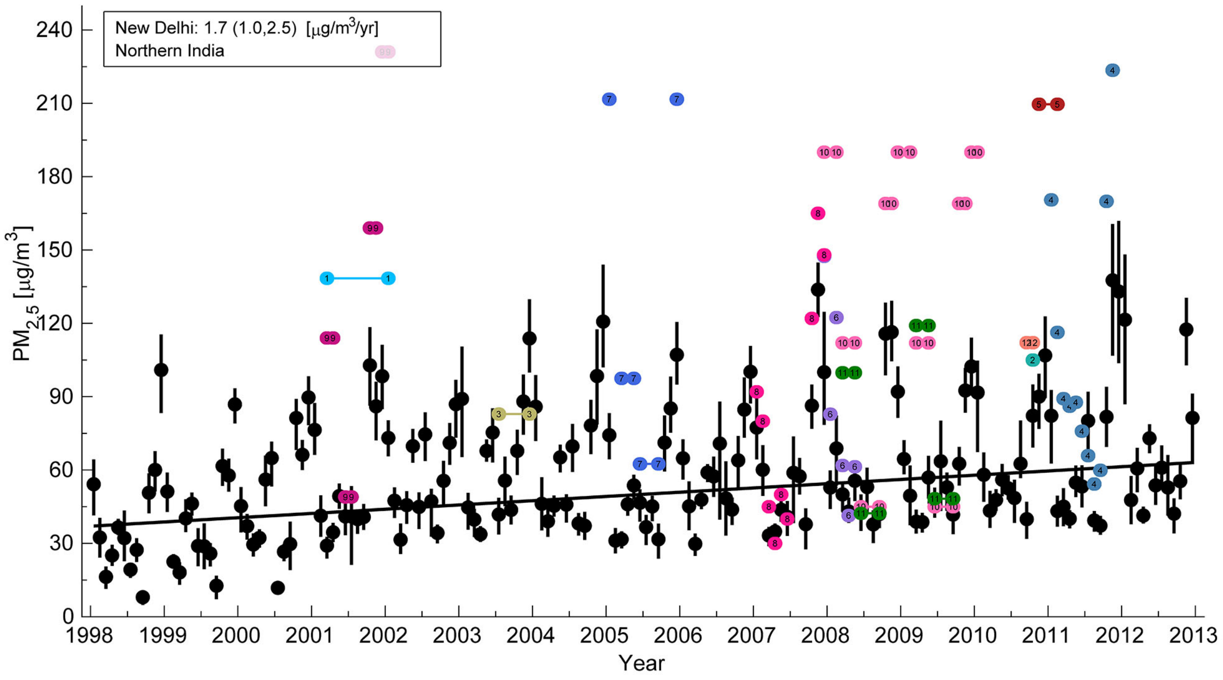

Figure S4. $PM_{2.5}$ time-series for New Delhi. Black dots and vertical lines denote monthly mean and 25th-75th percentile of satellite-derived values. Trend and 95% confidence intervals based on these values are provided in the inset. $PM_{2.5}$ values collected from the literature are plotted with each number and color corresponding to a different source. Specific colors and numbers are referenced below. Differences in instrumentation, methodology and site selection inhibit the use of these disparate values for trends, but are provided for comparative purposes. Horizontal lines correspond to the measurement duration.

Figure S4 Key

No.	Color	Years	Reference
1		2001–2002	Chowdhury 2004
2		2010	Kaushar et al. 2013
3		2003	Kumar et al. 2007
4		2011	Tiwari et al. 2013a
5		2010–2011	Tiwari et al. 2013b
6		2007–2008	Srivastava et al. 2012
7		2005	Tiwari et al. 2008
8		2007	Tiwari et al. 2009
9		2001–2002	World Bank 2004
10		2007–2010	Hyvarinen et al. 2010
11		2008–2009	Hyvarinen et al. 2011
12		2010	Bisht et al. 2013

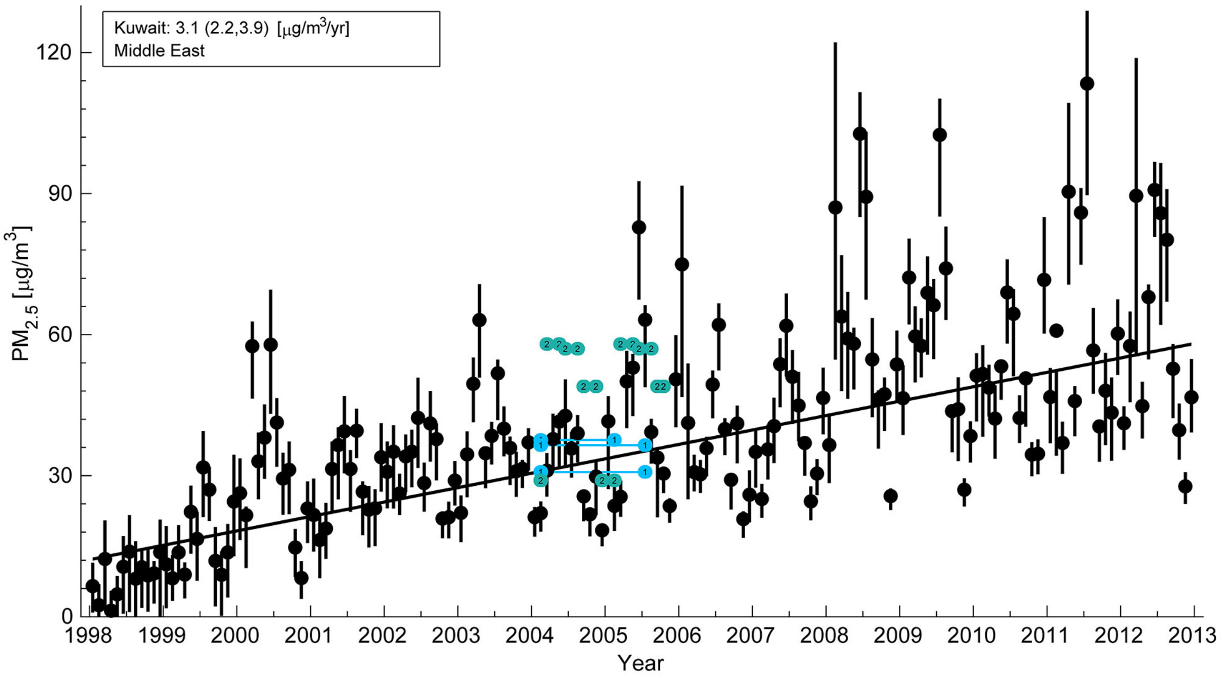

Figure S5. $PM_{2.5}$ time-series for Kuwait. Black dots and vertical lines denote monthly mean and 25th-75th percentile of satellite-derived values. Trend and 95% confidence intervals based on these values are provided in the inset. $PM_{2.5}$ values collected from the literature are plotted with each number and color corresponding to a different source. Specific colors and numbers are referenced below. Differences in instrumentation, methodology and site selection inhibit the use of these disparate values for trends, but are provided for comparative purposes. Horizontal lines correspond to the measurement duration.

Figure S5 Key

No.	Color	Years	Reference
1	● (light blue)	2004–2005	Brown et al. 2008
2	● (teal)	2004–2005	Alolayan et al. 2013

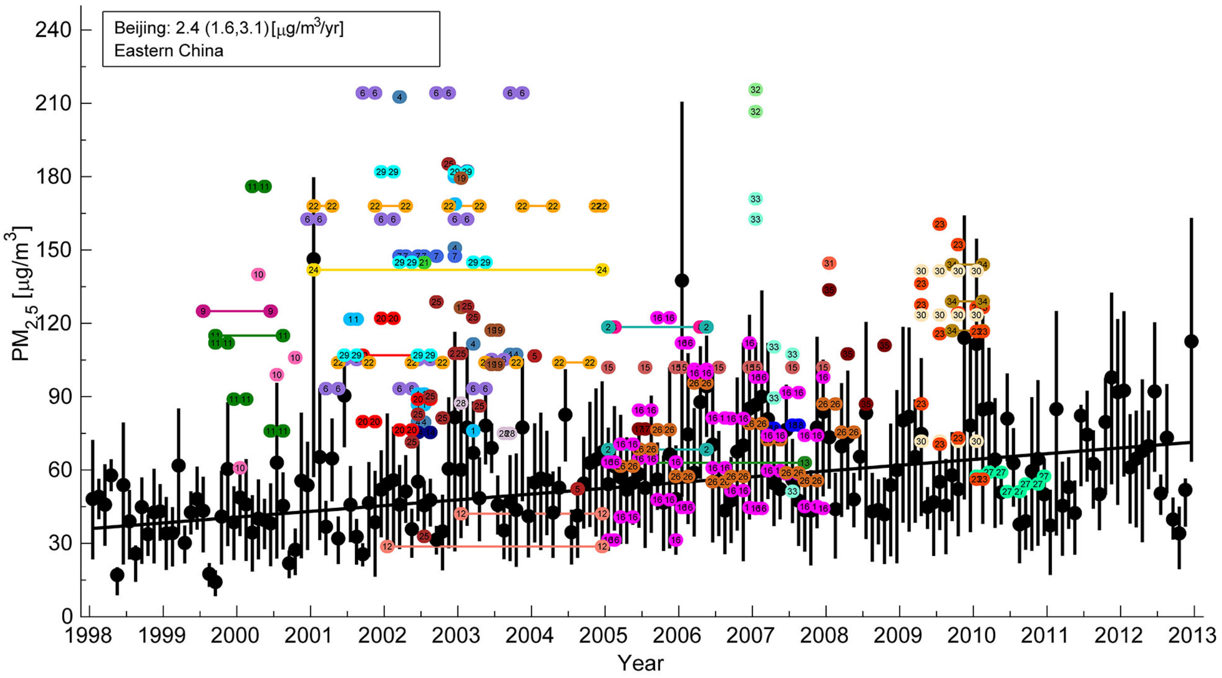

Figure S6. PM$_{2.5}$ time-series for Beijing. Black dots and vertical lines denote monthly mean and 25th-75th percentile of satellite-derived values. Trend and 95% confidence intervals based on these values are provided in the inset. PM$_{2.5}$ values collected from the literature are plotted with each number and color corresponding to a different source. Specific colors and numbers are referenced below. Differences in instrumentation, methodology and site selection inhibit the use of these disparate values for trends, but are provided for comparative purposes. Horizontal lines correspond to the measurement duration.

Figure S6 Key

No.	Color	Years	Reference
1		2001–2003	Dan et al. 2004
2		2005–2006	He et al. 2012
3		2005	Pathak et al. 2011
4		2002–2003	Han et al. 2005
5		2004	Song et al. 2007
6		2000–2003	Wang et al. 2005
7		2004	Wang et al. 2007
8		2005–2006	Yang et al. 2011
9		1999–2000	Yang et al. 2005
10		2000	Zheng et al. 2005
11		1999–2000	He et al. 2001
12		2002–2004	Hopke et al. 2008
13		2005–2007	Schleicher et al. 2011
14		2002–2003	Sun et al. 2004
15		2005–2007	Wang et al. 2009
16		2005–2007	Zhao et al. 2009
17		2005	Zhou et al. 2009
18		2007	Deng et al. 2011
19		2003	Cao et al. 2007
20		2001–2002	Duan et al. 2006
21		2002	He et al. 2004
22		2001–2004	Oanh et al. 2006
23		2009–2010	Zhao et al. 2013b
24		2001–2004	Zhang et al. 2007
25		2002–2003	Xu et al. 2005
26		2005–2008	Yu et al. 2011
27		2010	Yu et al. 2013
28		2003	Yu et al. 2005
29		2001–2003	Zhang et al. 2010
30		2009–2010	Zhao et al. 2013a
31		2008	Gu et al. 2011
32		2007	Li et al. 2009
33		2007	Li and Bai 2009
34		2009–2010	Li et al. 2012
35		2008	Gu et al. 2010

Table S1. Effect of seasonal variation on satellite-derived and ground-level $PM_{2.5}$ agreement over North America, 2001-2010. Mean and standard deviation of monthly mean agreements is given. The agreement of simulated seasonality applied to annual mean satellite-derived $PM_{2.5}$ is also given. Monthly values represent the center month of a three-month temporal range. Approximately 1000 locations are used.

Time Period	Satellite Seasonality: 1σ error [% + 1 μg/m^3]	Satellite Seasonality: Slope	Satellite Seasonality: Offset	Satellite Seasonality: Pearson Coefficient	Simulated Seasonality: 1σ error [% + 1 μg/m^3]	Simulated Seasonality: Slope	Simulated Seasonality: Offset	Simulated Seasonality: Pearson Coefficient
Annual	20 ± 2	1.07 ± 0.10	-1.4 ± 0.7	0.68 ± 0.07	20 ± 2	1.07 ± 0.10	-1.4 ± 0.7	0.68 ± 0.07
January	36 ± 3	1.47 ± 0.27	-4.9 ± 2.4	0.37 ± 0.06	26 ± 3	0.87 ± 0.11	0.6 ± 0.7	0.48 ± 0.04
February	33 ± 4	1.54 ± 0.23	-5.4 ± 2.2	0.45 ± 0.08	24 ± 2	0.91 ± 0.09	0.1 ± 0.7	0.57 ± 0.06
March	28 ± 3	1.49 ± 0.21	-4.7 ± 2.0	0.51 ± 0.08	20 ± 2	0.99 ± 0.10	-0.6 ± 0.8	0.65 ± 0.07
April	24 ± 3	1.30 ± 0.18	-2.6 ± 1.4	0.59 ± 0.06	19 ± 2	1.03 ± 0.13	-0.7 ± 0.9	0.66 ± 0.08
May	22 ± 3	1.24 ± 0.18	-2.3 ± 1.5	0.62 ± 0.09	21 ± 3	1.00 ± 0.13	-0.5 ± 0.9	0.62 ± 0.12
June	23 ± 3	1.23 ± 0.18	-2.7 ± 1.5	0.66 ± 0.12	23 ± 4	0.98 ± 0.12	-0.6 ± 1.0	0.64 ± 0.15
July	23 ± 3	1.24 ± 0.16	-3.2 ± 1.6	0.68 ± 0.11	24 ± 4	0.94 ± 0.08	-0.4 ± 0.9	0.68 ± 0.14
August	24 ± 4	1.14 ± 0.21	-2.3 ± 1.6	0.68 ± 0.11	22 ± 4	0.98 ± 0.10	-0.7 ± 0.8	0.71 ± 0.11
September	25 ± 5	1.06 ± 0.23	-1.5 ± 1.5	0.64 ± 0.10	21 ± 2	1.06 ± 0.16	-0.9 ± 1.0	0.69 ± 0.08
October	30 ± 5	0.94 ± 0.20	-0.7 ± 1.1	0.53 ± 0.10	24 ± 3	1.10 ± 0.17	-1.2 ± 1.1	0.60 ± 0.06
November	34 ± 5	0.95 ± 0.15	-0.7 ± 0.8	0.42 ± 0.08	27 ± 4	1.02 ± 0.11	-0.5 ± 0.7	0.49 ± 0.06
December	37 ± 4	1.05 ± 0.15	-1.3 ± 1.7	0.37 ± 0.07	27 ± 3	0.87 ± 0.10	0.6 ± 0.7	0.45 ± 0.05

Table S1 summarizes the variation in seasonal agreement between the satellite-derived and ground-based $PM_{2.5}$ at approximately 1000 locations in North America. Seasonal agreement varies with expected patterns of AOD retrieval accuracy, with improved agreement during summer months when surface reflectance is better characterized and when seasonal $PM_{2.5}$ enhancements increase the aerosol signal in satellite observations. We also provide the agreement found when applying GEOS-Chem seasonality to the satellite-derived annual means. Simulated seasonal variation improves monthly satellite-derived $PM_{2.5}$, particularly in the winter season when satellite retrievals can be inhibited by snow-cover. Seasonal cycles will vary globally, but these results suggest that the impact of snow, cloud and reduced sampling may increase the uncertainty of seasonal decadal mean $PM_{2.5}$ estimates by up to a factor of two relative to annual mean values.

Table S2. Effect of temporal range on satellite-derived and ground-level $PM_{2.5}$ agreement over North America. Mean and standard deviation of individual temporal comparisons are given (e.g. mean and standard deviation of annual agreement when temporal range is 1 year). Sites must be active for at least 80% of the temporal range, resulting in ca. 1000 locations used.

Temporal Range(yrs)	1σ error [% + 1 μg/m^3]	Slope	Offset	Pearson Coefficient
1	20 ± 2	1.07 ± 0.10	-1.4 ± 0.7	0.68 ± 0.07
2	17 ± 2	1.05 ± 0.08	-1.2 ± 0.7	0.72 ± 0.06
3	17 ± 2	1.04 ± 0.08	-1.2 ± 0.7	0.73 ± 0.05
4	15 ± 2	1.02 ± 0.06	-1.0 ± 0.5	0.74 ± 0.04
5	16 ± 1	1.02 ± 0.04	-1.0 ± 0.3	0.75 ± 0.03
6	15 ± 2	1.01 ± 0.04	-0.9 ± 0.3	0.77 ± 0.03
7	15 ± 2	1.00 ± 0.04	-0.8 ± 0.3	0.77 ± 0.03
8	14 ± 1	1.00 ± 0.03	-0.8 ± 0.2	0.78 ± 0.02
9	14 ± 1	1.00 ± 0.02	-0.8 ± 0.2	0.78 ± 0.02
10	14 ± 0	1.00 ± 0.00	-0.7 ± 0.0	0.79 ± N/A

Table S2 evaluates the impact of temporal range on accuracy, comparing mean satellite-derived and ground-based $PM_{2.5}$ over a varying number of years at ca. 1000 locations in North America. On average, annual performance is degraded significantly from decadal mean values (r=0.68 vs. r=0.79; slope=1.07 vs. 1.00; 1σ error = 20% vs. 14%). Errors in long-term exposure assessment increase with decreasing number of measurements from satellite. Sub-annual agreement of three-month running means further increases error by up to a factor of two. Significant improvement, however, is found when using as few as three years (r = 0.73; slope = 1.05; 1σ error = 17%), although still well below decadal agreement. As a result, the spatial correlations obtained over European and global regions (Figures 2 and 3) may indicate comparable significance to North America, only reduced by the limited sampling period of available ground-level observations for comparison.

References

Alexander, B, RJ Park, DJ Jacob, QB Li, RM Yantosca, J Savarino, et al. 2005. Sulfate formation in sea-salt aerosols: Constraints from oxygen isotopes. J. Geophys. Res. 110(D10).

Alolayan, MA, KW Brown, JS Evans, WS Bouhamra and P Koutralds. 2013. Source apportionment of fine particles in Kuwait City. Science of the Total Environment 448: 14–25.

Bisht, DS, S Tiwari, AK Srivastava and MK Srivastava. 2013. Assessment of air quality during 19th Common Wealth Games at Delhi, India. Natural Hazards 66(2): 141–154.

Boys, B, RV Martin, A Van Donkelaar, R MacDonell, NC Hsu, MJ Cooper, et al. 2014. Fifteen year global time series of satellite-derived fine particulate matter. Environ. Sci. Technol.

Brown, KW, W Bouhamra, DP Lamoureux, JS Evans and P Koutrakis. 2008. Characterization of particulate matter for three sites in Kuwait. Journal of the Air & Waste Management Association 58(8): 994–1003.

Cao, JJ, SC Lee, JC Chow, JG Watson, KF Ho, RJ Zhang, et al. 2007. Spatial and seasonal distributions of carbonaceous aerosols over China. J. Geophys. Res. 112(D22).

Chowdhury, M. 2004. Characterization of fine particle air pollution in the indian subcontinent. PhD. Dissertation, Georgia Institute of Technology.

Dan, M, GS Zhuang, XX Li, HR Tao and YH Zhuang. 2004. The characteristics of carbonaceous species and their sources in PM2.5 in Beijing. Atmos. Environ. 38(21): 3443–3452.

Deng, C, G Zhuang, K Huang, J Li, R Zhang, Q Wang, et al. 2011. Chemical characterization of aerosols at the summit of Mountain Tai in Central East China. Atmos. Chem. Phys. 11(14): 7319–7332.

Diner, DD, BH Braswell, R Davies, N Gobron, J Hu, Y Jin, et al. 2005. The value of multiangle measurements for retrieving structurally and radiatively consistent properties of clouds, aerosols, and surfaces. Remote Sens. Environ. 97: 495–518.

Duan, FK, KB He, YL Ma, FM Yang, XC Yu, SH Cadle, et al. 2006. Concentration and chemical characteristics of PM2.5 in Beijing, China: 2001-2002. Science of the Total Environment 355(1-3): 264–275.

Fairlie, TD, DJ Jacob and RJ Park. 2007. The impact of transpacific transport of mineral dust in the United States. Atmos. Environ. 41(6): 1251–1266.

Gu, JX, ZP Bai, WF Li, LP Wu, AX Liu, HY Dong, et al. 2011. Chemical composition of PM2.5 during winter in Tianjin, China. Particuology 9(3): 215–221.

Gu, JX, ZP Bai, AX Liu, LP Wu, YY Xie, WF Li, et al. 2010. Characterization of Atmospheric Organic Carbon and Element Carbon of PM2.5 and PM10 at Tianjin, China. Aerosol and air quality research 10(2): 167–176.

Han, LH, GS Zhuang, S Yele and ZF Wang. 2005. Local and non-local sources of airborne particulate pollution at Beijing. Science in China Series B-Chemistry 48(3): 253–264.

He, K, F Yang, Y Ma, Q Zhang, X Yao, CK Chan, et al. 2001. The characteristics of PM2.5 in Beijing, China. Atmos. Environ. 35(29): 4959–4970.

He, K, Q Zhao, Y Ma, F Duan, F Yang, Z Shi, et al. 2012. Spatial and seasonal variability of PM2.5 acidity at two Chinese megacities: insights into the formation of secondary inorganic aerosols. Atmos. Chem. Phys. 12(3): 1377–1395.

He, Z, YJ Kim, KO Ogunjobi, JE Kim and SY Ryu. 2004. Carbonaceous aerosol characteristics of PM2.5 particles in northeastern Asia in summer 2002. Atmos. Environ. 38(12): 1795–1800.

Henze, DK, JH Seinfeld, NL Ng, JH Kroll, TM Fu, DJ Jacob, et al. 2008. Global modeling of secondary organic aerosol formation from aromatic hydrocarbons: high- vs. low-yield pathways. Atmos. Chem. Phys. 8: 2405–2421.

Holben, BN, D Tanre, A Smirnov, TF Eck, I Slutsker, N Abuhassan, et al. 2001. An emerging ground-based aerosol climatology: Aerosol optical depth from AERONET. J. Geophys. Res. 106(D11): 12067–12097.

Hopke, PK, DD Cohen, BA Begum, SK Biswas, BF Ni, GG Pandit, et al. 2008. Urban air quality in the Asian region. Science of the Total Environment 404(1): 103–112.

Hsu, NC, R Gautam, AM Sayer, C Bettenhausen, C Li, MJ Jeong, et al. 2012. Global and regional trends of aerosol optical depth over land and ocean using SeaWiFS measurements from 1997-2010. Atmos. Chem. Phys. 12: 8037–8053.

Hsu, NC, MJ Jeong, C Bettenhausen, AM Sayer, R Hansell, CS Seftor, et al. 2013. Enhanced Deep Blue aerosol retrieval algorithm: The second generation. J. Geophys. Res. 118: 1–20.

Hyvarinen, AP, H Lihavainen, M Komppula, TS Panwar, VP Sharma, RK Hooda, et al. 2010. Aerosol measurements at the Gual Pahari EUCAARI station: preliminary results from in-situ measurements. Atmos. Chem. Phys. 10(15): 7241–7252.

Hyvarinen, AP, T Raatikainen, D Brus, M Komppula, TS Panwar, RK Hooda, et al. 2011. Effect of the summer monsoon on aerosols at two measurement stations in Northern India - Part 1: PM and BC concentrations. Atmos. Chem. Phys. 11(16): 8271–8282.

Kahn, RA, BJ Gaitley, JV Martonchik, DJ Diner, KA Crean and B Holben. 2005. Multiangle Imaging Spectroradiometer (MISR) global aerosol optical depth validation based on 2 years of coincident Aerosol Robotic Network (AERONET) observations. J. Geophys. Res. 110(D10).

Kaushar, A, D Chate, G Beig, R Srinivas, N Parkhi, T Satpute, et al. 2013. Spatio-Temporal Variation and Deposition of Fine and Coarse Particles during the Commonwealth Games in Delhi. Aerosol and air quality research 13(2): 748–755.

Kumar, N, A Chu and A Foster. 2007. An empirical relationship between PM2.5 and aerosol optical depth in Delhi Metropolitan. Atmos. Environ. 41(21): 4492–4503.

Levy, RC, LA Remer and O Dubovik. 2007. Global aerosol optical properties and application to Moderate Resolution Imaging Spectroradiometer aerosol retrieval over land. J. Geophys. Res. 112(D13210).

Li, PH, B Han, J Huo, B Lu, X Ding, L Chen, et al. 2012. Characterization, Meteorological Influences and Source Identification of Carbonaceous Aerosols during the Autumn-winter Period in Tianjin, China. Aerosol and air quality research 12(2): 283–294.

Li, WF and ZP Bai. 2009. Characteristics of organic and elemental carbon in atmospheric fine particles in Tianjin, China. Particuology 7(6): 432–437.

Li, WF, ZP Bai, AX Liu, J Chen and L Chen. 2009. Characteristics of Major PM2.5 Components during Winter in Tianjin, China. Aerosol and air quality research 9(1): 105–119.

Martonchik, JV, DJ Diner, KA Crean and MA Bull. 2002. Regional aerosol retrieval results from MISR. IEEE T. Geosci. Remote 40(7): 1520–1531.

Martonchik, JV, RA Kahn and DJ Diner. 2009. Retrieval of Aerosol Properties over Land Using MISR Observations. In: Satellite Aerosol Remote Sensing Over Land, (AA Kokhanovsky and Gd Leeuw, eds). Berlin:Springer, 267–293.

Oanh, NTK, N Upadhyaya, YH Zhuang, ZP Hao, DVS Murthy, P Lestari, et al. 2006. Particulate air pollution in six Asian cities: Spatial and temporal distributions, and associated sources. Atmos. Environ. 40(18): 3367–3380.

Park, RJ, DJ Jacob, M Chin and RV Martin. 2003. Sources of carbonaceous aerosols over the United States and implications for natural visibility. J. Geophys. Res. 108(D12).

Park, RJ, DJ Jacob, BD Field, RM Yantosca and M Chin. 2004. Natural and transboundary pollution influences on sulfate-nitrate-ammonium aerosols in the United States: Implications for policy. J. Geophys. Res. 109(D15).

Pathak, RK, T Wang, KF Ho and SC Lee. 2011. Characteristics of summertime PM2.5 organic and elemental carbon in four major Chinese cities: Implications of high acidity for water-soluble organic carbon (WSOC). Atmos. Environ. 45(2): 318–325.

Remer, LA, RG Kleidman, RC Levy, YJ Kaufman, D Tanre, S Mattoo, et al. 2008. Global aerosol climatology from the MODIS satellite sensors. J. Geophys. Res. 113(D14).

Sayer, AM, NC Hsu, C Bettenhausen, M-J Jeong and J Zhang. 2012. Global and regional evaluation of over-land spectral aerosol optical depth retrievals from SeaWiFS. Atmos. Meas. Tech. 5: 1761–1778.

Schleicher, NJ, S Norra, FH Chai, YZ Chen, SL Wang, KQ Cen, et al. 2011. Temporal variability of trace metal mobility of urban particulate matter from Beijing - A contribution to health impact assessments of aerosols. Atmos. Environ. 45(39): 7248–7265.

Song, Y, XY Tang, S Xie, Y Zhang, Y Wei, M Zhang, et al. 2007. Source apportionment of PM2.5 in Beijing in 2004. J. Harzardous Materials 146: 124–130.

Srivastava, AK, S Singh, S Tiwari, VP Kanawade and DS Bisht. 2012. Variation between near-surface and columnar aerosol characteristics during the winter and summer at Delhi in the Indo-Gangetic Basin. Journal of Atmospheric and Solar-Terrestrial Physics 77: 57–66.

Sun, Y, G Zhuang, Y Wang, L Han, J Guo, M Dan, et al. 2004. The air-borne particulate pollution in Beijing--concentration, composition, distribution and sources. Atmos. Environ. 38(35): 5991–6004.

Tiwari, S, AK Srivastava, DS Bisht, T Bano, S Singh, S Behura, et al. 2009. Black carbon and chemical characteristics of PM10 and PM2.5 at an urban site of North India. Journal of Atmospheric Chemistry 62(3): 193–209.

Tiwari, S, AK Srivastava, DS Bisht, P Parmita, MK Srivastava and SD Attri. 2013a. Diurnal and seasonal variations of black carbon and PM2.5 over New Delhi, India: Influence of meteorology. Atmospheric Research 125: 50–62.

Tiwari, S, AK Srivastava, DS Bisht, PD Safai and P Parmita. 2013b. Assessment of carbonaceous aerosol over Delhi in the Indo-Gangetic Basin: characterization, sources and temporal variability. Natural Hazards 65(3): 1745–1764.

Tiwari, S, MK Srivastava and DS Bisht. 2008. Chemical Characteristics of Water Soluble Components of Fine Particulate Matter, PM2.5, at Delhi, India. e-Journal Earth Science India 1(3): 72–86.

Torseth, K, W Aas, K Breivik, AM Fjaeraa, M Fiebig, AG Hjellbrekke, et al. 2012. Introduction to the European Monitoring and Evaluation Programme (EMEP) and observated atmosperhic composition change during 1972-2009. Atmos. Chem. Phys. 12: 5447–5481.

van Donkelaar, A, RV Martin, M Brauer, R Kahn, R Levy, C Verduzco, et al. 2010. Global Estimates of Ambient Fine Particulate Matter Concentrations from Satellite-Based Aerosol Optical Depth: Development and Application. Environ. Health Perspect. 118(6): 847–855.

van Donkelaar, A, RV Martin, RJD Spurr, E Drury, LA Remer, RC Levy, et al. 2013. Optimal estimation for global ground-level fine particulate matter concentrations. J. Geophys. Res. 118: 1–16.

Wang, HL, YM Zhou, YH Zhuang, XK Wang and ZP Hao. 2009. Characterization of PM(2.5)/PM(2.5-10) and source tracking in the juncture belt between urban and rural areas of Beijing. Chinese Science Bulletin 54(14): 2506–2515.

Wang, Y, G Zhuang, A Tang, H Yuan, Y Sun, S Chen, et al. 2005. The ion chemistry and the source of PM2.5 aerosol in Beijing. Atmos. Environ. 39: 3771–3784.

Wang, Y, GS Zhuang, S Chen, ZS An and AH Zheng. 2007. Characteristics and sources of formic, acetic and oxalic acids in PM2.5 and PM10 aerosols in Beijing, China. Atmospheric Research 84(2): 169–181.

World Bank. 2004. Toward Cleaner Urban Air in South Asia: Tackling Transport Pollution, Understanding Sources.The World Bank.

Xu, DD, M Dan, Y Song, ZF Chai and GS Zhuang. 2005. Concentration characteristics of extractable organohalogens in PM2.5 and PM10 in Beijing, China. Atmos. Environ. 39(22): 4119–4128.

Yang, F, J Tan, Q Zhao, Z Du, K He, Y Ma, et al. 2011. Characteristics of PM2.5 speciation in representative megacities and across China. Atmos. Chem. Phys. 11(11): 5207–5219.

Yang, FM, BM Ye, KB He, YL Ma, SH Cadle, T Chan, et al. 2005. Characterization of atmospheric mineral components of PM2.5 in Beijing and Shanghai, China. Science of the Total Environment 343(1-3): 221–230.

Yu, JH, B Guinot, T Yu, X Wang and WQ Liu. 2005. Seasonal variations of number size distributions and mass concentrations of atmospheric particles in Beijing. Advances in Atmospheric Sciences 22(3): 401–407.

Yu, LD, GF Wang, RJ Zhang, LM Zhang, Y Song, BB Wu, et al. 2013. Characterization and Source Apportionment of PM2.5 in an Urban Environment in Beijing. Aerosol and air quality research 13(2): 574–583.

Yu, Y, N Schleicher, S Norra, M Fricker, V Dietze, U Kaminski, et al. 2011. Dynamics and origin of PM2.5 during a three-year sampling period in Beijing, China. Journal of environmental monitoring 13(2): 334–346.

Zhang, J and JS Reid. 2010. A decadal regional and global trend analysis of the aerosol optical depth using a data-assimilation grade over-water MODIS and Level 2 MISR aerosol products. Atmos. Chem. Phys. 10: 10949–10963.

Zhang, W, JH Guo, YL Sun, H Yuan, GS Zhuang, YH Zhuang, et al. 2007. Source apportionment for,urban PM10 and PM2.5 in the Beijing area. Chinese Science Bulletin 52(5): 608–615.

Zhang, WJ, GS Zhuang, JH Guo, DQ Xu, W Wang, D Baumgardner, et al. 2010. Sources of aerosol as determined from elemental composition and size distributions in Beijing. Atmospheric Research 95(2-3): 197–209.

Zhao, PS, F Dong, D He, XJ Zhao, XL Zhang, WZ Zhang, et al. 2013a. Characteristics of concentrations and chemical compositions for PM2.5 in the region of Beijing, Tianjin, and Hebei, China. Atmos. Chem. Phys. 13(9): 4631–4644.

Zhao, PS, F Dong, YD Yang, D He, XJ Zhao, WZ Zhang, et al. 2013b. Characteristics of carbonaceous aerosol in the region of Beijing, Tianjin, and Hebei, China. Atmos. Environ. 71: 389–398.

Zhao, XJ, XL Zhang, XF Xu, J Xu, W Meng and WW Pu. 2009. Seasonal and diurnal variations of ambient PM2.5 concentration in urban and rural environments in Beijing. Atmos. Environ. 43(18): 2893–2900.

Zheng, M, LG Salmon, JJ Schauer, LM Zeng, CS Kiang, YH Zhang, et al. 2005. Seasonal trends in PM2.5 source contributions in Beijing, China. Atmos. Environ. 39(22): 3967–3976.

Zhou, XH, J Cao, T Wang, WS Wu and WX Wang. 2009. Measurement of black carbon aerosols near two Chinese megacities and the implications for improving emission inventories. Atmos. Environ. 43(25): 3918–3924.
